# Effect of Addition of Phosphate of Lime to Food of Lambs

**Published:** 1874-07

**Authors:** 


### EFFECT OF ADDITION OF PHOSPHATE OF LIME TO FOOD OF LAMBS.

NUMEROUS comparative experiments to solve the above problem were made by Dr. Hofmeister, by feeding to one lot of lambs, six weeks old, hay and potatoes alone, and to another phosphate of lime in addition. Although the phosphate diet seemed to produce a better appetite, yet, since greater increase in weight did not seem to accompany increased consumption of food, it was concluded that, at least by the moderate feeding employed, no peculiar effect in increasing the live weight could be ascribed to phosphate of lime. The ratio of live weight to dead weight was found upon slaughtering to be the same in both cases. The bones of those not treated with phosphate were, for the most part, richer in fat, but the phosphate seemed to be without perceptible effect upon the amount of lime or phosphoric acid in the bones. The experiments tend to substantiate the opinion of Haubner, that the addition of phosphate of lime has no influence upon the formation of bone or flesh, unless the usual food is actually deficient in it. The animals treated with phosphate digested on an average 37.5 per cent. more phosphoric acid and 23.8 per cent. more lime than the others. Experiments with sheep two years old with superphosphate of lime showed that with 150 grains administered per day, 51.1 per cent. of its phosphoric acid and 41.7 per cent. of its lime were digested; and of 300 grains, 31.6 per cent. of its phosphoric acid and 64.71 per cent. of its lime were digested. No injurious effects were perceived.

